# An Immunomodulating Peptide with Potential to Promote Anticancer Immunity Without Compromising Immune Tolerance

**DOI:** 10.3390/biomedicines13081908

**Published:** 2025-08-05

**Authors:** Michael Agrez, Christopher Chandler, Amanda L. Johnson, Marlena Sorensen, Kirstin Cho, Stephen Parker, Benjamin Blyth, Darryl Turner, Justyna Rzepecka, Gavin Knox, Anastasia Nika, Andrew M. Hall, Hayley Gooding, Laura Gallagher

**Affiliations:** 1 InterK Peptide Therapeutics Limited, Sydney, NSW 2113, Australia; 2 Auspep Pty Limited, Melbourne, VIC 3043, Australia; chris@auspep.com.au; 3 Inotiv Inc., Boulder, CO 80301, USA; amanda.johnson@inotiv.com (A.L.J.); marlena.sorensen@inotiv.com (M.S.);; 4 Models of Cancer Translational Research Centre, Research Division, Peter MacCallum Cancer Centre, 305 Grattan St., Melbourne, VIC 3000, Australia; benjamin.blyth@petermac.org; 5 Sir Peter MacCallum Department of Oncology, The University of Melbourne, Parkville, VIC 3010, Australia; 6 Concept Life Sciences, Edinburgh EH16 4UX, UK; darryl.turner@conceptlifesciences.com (D.T.); justyna.rzepecka@conceptlifesciences.com (J.R.); gavin.knox@conceptlifesciences.com (G.K.); anastasia.nika@conceptlifesciences.com (A.N.); andrew.hall@conceptlifesciences.com (A.M.H.); hayley.gooding@conceptlifesciences.com (H.G.); lauragallagher99@gmail.com (L.G.)

**Keywords:** peptide, anticancer immunity, immune tolerance, cytokines, signalling

## Abstract

**Background:** Immune checkpoint inhibitor therapy in patients with lung cancer and metastatic melanoma is associated with exacerbation of autoimmune-related diseases. The efficacy of treatment targeting the programmed cell death receptor-1 (PD-1) checkpoint relies upon a feedback loop between interferon gamma (IFN-γ) and the interleukin-12 isoform, IL-12p40. Paradoxically, both cytokines and the anti-PD-1 antibody worsen psoriasis. We previously reported an immunomodulating peptide, designated IK14004, that inhibits progression of Lewis lung cancer in mice yet uncouples IFN-γ from IL-12p40 production in human immune cells. **Methods**: Immune cells obtained from healthy donors were exposed to IK14004 in vitro to further characterise the signalling pathways affected by this peptide. Using C57BL/6 immunocompetent mice, the effect of IK14004 was tested in models of lung melanoma and psoriatic skin. **Results**: Differential effects of IK14004 on the expression of IFN-α/β, the interleukin-15 (IL-15) receptor and signal transducers and activators of transcription were consistent with immune responses relevant to both cancer surveillance and immune tolerance. Moreover, both melanoma and psoriasis were inhibited by the peptide. **Conclusions**: Taken together, these findings suggest mechanisms underlying immune homeostasis that could be exploited in the setting of cancer and autoimmune pathologies. Peptide administered together with checkpoint blockers in relevant models of autoimmunity and cancer may offer an opportunity to gain further insight into how immune tolerance can be retained in patients receiving cancer immunotherapy.

## 1. Introduction

Melanoma and autoimmune comorbidities are linked [1], and autoimmune pathologies are exacerbated by cancer immunotherapy [2]. This has been highlighted by immune checkpoint inhibitor (ICI) therapy that targets the programmed cell death receptor-1 (PD-1) checkpoint [3]. Under physiological conditions, presentation of low-affinity self-peptides by immature dendritic cells (DCs) activates PD-1-mediated signalling which maintains immune tolerance by ensuring that immune responses are not generated against chronically present autoantigens [4]. However, immune-related adverse events (irAEs) occur in more than 50% of melanoma and lung cancer patients treated with the anti-PD-1 antibody [3]. Moreover, the immune system needs to be activated in such a way that tolerance for self-antigens is maintained, and unfortunately immunomodulatory drugs used in the management of irAEs can lead to tumour escape [5].

Immunosuppressive T regulatory cells (Tregs) prevent excessive immune reactions to self-antigens by autoreactive T cells [6] and can inhibit every cell type including natural killer cells [7]. However, PD-1 signalling and Tregs dampen the magnitude of immune responses against cancer-related “foreign antigens” upon contact between effector T (Teff) cells and mature DCs. Hence, targeting either immune checkpoints or Tregs are important strategies in cancer immunotherapy [8,9] notwithstanding the considerable risk of irAEs.

The effects of interleukin-12 (IL-12) on anticancer immunity and immune tolerance are complex. The IL-12 heterodimer, IL-12p70, comprises a p35 subunit linked to a p40 subunit. The homo-dimeric IL-12p40 isoform is produced in excess compared with IL-12p70 [10] and suppresses Treg function [11] in contrast to 12p70 which promotes induction of Th1-like Tregs [12]. Production of IL-12 by mature DCs regulates interferon-gamma (IFN-γ) production by pro-inflammatory Th1-differentiated T cells [13], and DC maturation varies with IL-12-producing capacity [14].

Interleukin-12-stimulated natural killer (NK) cells can also be primed by pre-stimulation with interleukin-15 (IL-15) leading to enhanced phosphorylation of STAT4 upon IL-12 stimulation [15]. IL-15 is the most potent soluble mediator enabling NK cell maturation and activation [15]. A variety of cell types produce IL-15 and present it in *trans* to NK cells together with the high-affinity IL-15α (CD215) receptor subunit [16]. The virtual absence of natural killer (NK) cells in IL-15α-deficient mice indicates that IL-15 receptor signals are critical for NK cell development [17], and under steady state conditions, the survival of NK cells is maintained by lymphoid tissue-derived IL-15 [16]. Unlike interleukin-2 (IL-2), IL-15 does not enhance Treg cell functionality [18].

However, manipulating IL-2 and its receptor can dramatically shift the balance between IL-2-producing effector T (Teff) cells and IL-2-responsive Tregs [19]. This is highlighted by the observation that low dose IL-2 significantly improves the clinical manifestations of psoriasis associated with amplification of the percentage of immunosuppressive Tregs [20]. At high doses, the therapeutic effect of IL-2 in the treatment of cancer reflects its ability to stimulate proliferation of cytotoxic CD8+ T calls and NK cells [21]. For example, IL-2 primes NK cells by upregulating the expression of the β2 signalling chain of the IL-12p70 receptor complex (IL-12Rβ2) which is not only essential for NK cell lysis activity [22] but also dependent upon the signal transducer and activator of transcription-4 (STAT4) [23]. We have reported that a lipidic peptide, designated IK14004, exerts effects that may contribute to both immune tolerance and anticancer immunity. For example, in human immune cells the peptide enhances IL-2 production and expansion of Tregs [24] while also inducing expression of IL-12Rβ2 in NK cells [25].

In addition, Type I IFNs are involved in both cancer and autoimmunity. For example, in systemic autoimmune disorders such as rheumatoid arthritis, the IFN-α:IFN-β ratio may be critical with a lower ratio perceived as beneficial [26]. IFN-α has been implicated in SLE [27] and the exacerbation of psoriasis [28]. In contrast, IFN-β induces proliferation of Tregs [29,30] and promotes Treg-mediated suppression of conventional Teff cell proliferation [31]. Hence, IFN-β has been used to treat autoimmune diseases such as Behcet’s syndrome and multiple sclerosis (MS) [27]. Moreover, in the largest epidemiological study of MS patients reported, administration of IFN-β has not been thought to play a role in the pathogenesis of psoriasis [32]. This may be due, in part at least, because IFN-β suppresses IL-17 production by inhibiting activation of STAT3 in CD4+ T cells [33]. Notably, IFN-β also inhibits the growth of melanoma cells by induction of cross-priming of CD8+ T cells by DCs in vitro and in vivo [34], and its antitumour effects against melanoma are generally better than IFN-α [35].

Successful anti-PD-1 cancer immunotherapy requires crosstalk between DCs and T cells with IL-12p40 being produced by DCs upon sensing interferon-gamma (IFN-γ) released from neighbouring T cells [36]. However, a paradox exists in so far as IFN-γ and IL-12p40 are critical for the preservation of cancer immunity in the presence of anti-PD-1 therapy on the one hand [36], while on the other hand autoimmune pathologies such as psoriasis [37,38] and systemic lupus erythematosus (SLE) [39] are linked to the presence of these cytokines. For example, expression of IFN-γ in serum and in the skin of psoriasis patients correlates with disease severity [37,40] whereas anti-IL-12p40 neutralising antibody (mAb) lessens disease activity in psoriasis patients [38]. Moreover, IL-12p70 activates STAT4 [41] which is thought not to be involved in the pathogenesis of psoriasis in contrast to STAT3 which acts as a key player in the psoriasis-inducing interleukin-23/interleukin-17 (IL-23/IL-17) axis [42]. Interestingly, IK14004 inhibits production of both IFN-γ and IL-12p40 in human immune cells in vitro as well as Lewis lung cancer (LLC) growth in vivo [24,25]. In the present study we extended our previously published in vitro findings in human immune cells and sought to determine whether the peptide could suppress both melanoma and psoriasis in murine models.

## 2. Materials and Methods

### 2.1. Ethics Approval

The human and animal studies were supported by the respective Committees, the details of which are given below. Written consent was obtained from all human participants.

Human ethics approval is as follows: All methods were carried out in accordance with relevant guidelines and regulations. Buffy coat samples from healthy human donors were obtained from Research Donors Limited via Cambridge BioScience:

Ethics Committee Name: Black County Research Committee;

Approval Code: 19/WM/0260;

Approval Date: 23 August 2019.

Animal ethics approval is as follows: The melanoma study was carried out in accordance with relevant guidelines and regulations as stipulated in the ARRIVE website (https://arriveguidelines.org):

Ethics Committee Name: Peter MacCallum Cancer Centre Animal Experimentation Ethics Committee;

Approval Code: E592;

Approval Date: 27 July 2017;

Animal ethics is as follows: The Imiquimod psoriasis study (IMQ-P) was performed by Inotiv, Boulder, CO, USA and was conducted in accordance with the test facility standard operating procedures (SOPs), the World Health Organization Quality Practices in Basic Biomedical Research guidelines, and in compliance with all state and federal regulations, including USDA Animal Welfare Act 9 CFR Parts 1–3, Federal Register 39129, 22 July 1993. No acceptable alternative test systems were identified for the animals used in this project:

Ethics Committee Name: IACUC—Institutional Animal Care and Use Committee;

Approval Code: IB-042;

Approval Date: 22 August 2023.

### 2.2. Peptide Synthesis

In brief, IK14004 was synthesized using solid phase peptide synthesis with Fmoc-protected amino acid building blocks. Four (2)Adod [(S)-2-aminododecanoic acid] residues (Watanabe Chemical Industries Ltd., Hiroshima Japan) were first coupled sequentially onto a Rink AM resin. Then the amino acid sequence RSKAKNPLYR was assembled by sequentially coupling each of the protected amino acids, in turn, onto the -(2)Adod-(2)Adod-(2)Adod-(2)Adod-amide resin. Once synthesis was complete the lipidic peptide was globally deprotected and cleaved from the resin liberating the crude, C-terminally amidated lipidic product. This was purified to 99% and salt exchanged to the acetate salt by RP-HPLC (C18). Supplementary Figure S1: Refers to structure, purity and amino acid analysis of IK14004.

### 2.3. Isolation and In Vitro Culture of Human PBMC and Immune Cell Subsets for Functional Analysis

Human peripheral blood mononuclear cells (PBMCs) were prepared from buffy coat samples using SepMate tubes, EasySep selection and enrichment kits, Lymphoprep, RoboSep Buffer and EasySep magnets (STEMCELL Technologies Inc., Cambridge, MA, USA). PBMCs were resuspended in RPMI-10 (RPMI-1640; ThermoFisher) supplemented with 10% heat inactivated Foetal Bovine Serum (LabTech, Tampa, FL, USA), 100 U/mL penicillin, 100 µg/mL streptomycin (ThermoFisher), 2 mM L-glutamine (ThermoFisher), and 50 µM β-mercaptoethanol (ThermoFisher) at 1 × 10^6^ cells/mL and plated at a density of 1 × 10^5^ per well (100 µL) in 96-well, flat-bottom culture plates. PBMCs were stimulated with 1 µg/mL of soluble anti-CD3 (BioLegend) and cultured for either 24 or 72 h at 37 °C and 5% CO_2_.

CD3+ T cells and CD3- Tcells/CD56+^dim^ NK cells were isolated from PBMCs by negative selection using immune-magnetic separation (Stem cell kits). CD3+ T cells were resuspended in complete medium as used for PBMCs at 0.5 × 10^6^/mL and plated at a density of 5 × 10^4^ per well (100 µL) in 96-well, flat-bottom culture plates. The T cell preparations were stimulated with anti-CD3/anti-CD28-coated Dynabeads (ThermoFisher) at a 4:1 cell:bead ratio (1.25 × 10^4^/well; 50 µL volume) and cultured for 72 h. Isolated, non-stimulated NK cells were resuspended in complete medium as used for PBMCs at 0.25 × 10^6^/mL and plated at a density of 2.5 × 10^4^ per well (100 µL) and cultured for either 24 or 72 h.

The lipidic peptide, IK14004, was solubilised as a 1 mM stock solution in sterile milliQ water (Lonza, Basel, Switzerland) and added to wells at a final volume of 50 µL per well. Peptide concentrations ranged from 0.08 to 1.25 µM. Vehicle controls in peptide-based experiments comprised 0.13% sterile milliQ water in culture medium.

### 2.4. Flow Cytometry

Flow cytometry was performed on blood samples obtained only from human donors, and each donor’s sample was assessed separately. Peripheral blood mononuclear cells (PBMCs), CD3+ T cells and isolated NK cells were cultured for either 24 or 72 h prior to cell staining. Staining was performed to determine cell viability (Flexible Viability Dye eFluor^TM^ 780; ThermoFisher, Waltham, MA, USA) and expression of extracellular/intracellular markers using fluorescently labelled antibodies against the following human proteins: CD4 (FITC Mab OKT4; ThermoFisher); CD8 (BV711/clone SK1, BioLegend, San Diego, CA, USA); IL-2Rβ (CD122) (BioLegend, Cat. No. 339007) and IL-15α (CD215) (PE anti-human CD215, BioLegend, Cat. No. 330108). For intracellular staining, Brefeldin (3 μg/mL) (Life Technologies, Cat #00-4506-51, Lot #2229153, Carlsbad, CA, USA) was added to cultures four hours prior to flow cytometry. Intracellular expression of IFN-α and IFN-β in CD4+ T/CD8+ T/NK cells within PBMC cultures was determined using anti-IFN-α/β antibodies (PE; BD Bioscience and FITC BioTeche, respectively) as previously reported [24].

CD3+ T cells were fixed using the BD Phosflow™ Fix buffer I (BD Bioscience, Franklin Lakes, NJ, USA) and permeabilised to allow for intracellular staining using BD Phosflow™ Perm Buffer III (BD Bioscience). Cells were then stained with fluorochrome conjugated antibodies to detect phospho-STAT3 (PE anti-STAT3, pY705, Lot #562072, BD Bioscience) and phospho-STAT4 (eBioscience, Lot# 12-9044, San Diego, CA, USA). In STAT4 assays, CD3+ T cell cultures were also exposed to the Lck inhibitor A-770041 (Sigma-Aldrich, St. Louis, MO, USA) (100 nM in 0.1% DMSO *v*/*v*) for the duration of experiments in the absence/presence of IK14004. In experiments using unstimulated, isolated NK cells, expression of pSTAT4 and CD215 was determined after 72 h.

Each tissue culture experiment was performed using triplicate wells (technical replicates). Flow cytometry profiles are shown as either percentage of cells stained for a particular marker or level of marker expression (MFI). Dot plot and gating strategies are shown in the Supplementary Figures S2–S13.

### 2.5. ELISA Assays

Culture supernatants were obtained from anti-CD3-stimulated PBMCs and anti-CD3-/anti-CD28-stimulated T cells plated in triplicate after either 24 or 72 h. Production of IL-12p70/IL-15 and IL-23/TNF-α was determined using ELISA kits as follows: IL-12p70 (88-7162, ThermoFisher, Waltham, MA, USA); IL-15 (B228645, BioLegend, San Diego, CA, USA); IL-23, 213597003, ThermoFisher, Waltham, MA, USA) and TNF-α (264515-005, ThermoFisher, Waltham, MA, USA). ELISA plates were read at 450 nm using an Infinite F50 absorbance reader with Magellan™ data analysis software (Tecan Group Ltd., Mannedorf, Switzerland).

### 2.6. Proliferation Assay Comparing Effects of Peptide and Doxorubicin on B16F10 Melanoma Cell Growth In Vitro

B16F10 melanoma cells (sourced from the American Type Culture Collection, Manassas, VA, USA) were seeded into 96-well plates (1000 cells/well) in complete cell culture medium and allowed to attach for 24 h (37 °C, 5% CO_2_ in air). Next, an equal volume of either cell culture medium only, or 2 × concentration of drug dissolved in cell culture medium, was added to each of the 5 replicate wells (technical replicates) to expose cells to concentrations of IK14004 in the dose range from 0 to 5 µM. The chemotherapeutic agent, Doxorubicin, inhibits melanoma cell proliferation directly in contrast to the peptide which has potential to suppress cancer growth indirectly via its immunomodulating effects [25]. Hence, Doxorubicin was included as a positive control to confirm that at comparable concentrations the peptide was ineffective against B16F10 melanoma cells as has been reported previously for Lewis lung cancer cells [25]. Following cell attachment, the cells were cultured for 72 h in the presence of either IK14004 or Doxorubicin (2.5 µM). The cell culture medium was then removed, and the attached cells fixed in ice-cold trichloroacetic acid. Fixed cells were stained with Sulforhodamine B (SRB) and then washed with 1% acetic acid to remove unbound dye. The retained dye was solubilised in 10 mM Tris base solution, and the absorbance at 550 nm was measured with the baseline (media only without cells) subtracted. The data were normalised between the maximum proliferation (100%, cells with no drug) and the starting cell density (0%, cells before addition of drug). Each experiment was performed on two independent occasions (biological replicates).

### 2.7. Murine Melanoma Study

The study was performed at the Peter MacCallum Cancer Centre, Melbourne, Australia. Twenty-four female C57Bl/6 mice (WEHI, aged 8 weeks) were inoculated intravenously with 1 × 10^5^ B16F10 cells (passage number 12) in PBS on 8/05/2017 (Day 1). Mice were randomised into three groups of 8 mice before being injected intraperitoneally (IP) with 0.1 mL H_2_O or IK14004 (40 µg or 400 µg/mouse). The peptide was administered twice weekly for two weeks, and mice were monitored daily for general health during that time. The effects of twice weekly IP dosing with either H_2_O or 40 µg and 400 µg IK14004 in B16F10-bearing C57Bl/6 mice were also assessed by measuring mouse body weight and the mean percent weight change from baseline (Day 1) recorded, including SEM. The scoring of lung tumour nodules was performed by one individual blinded to the treatment groups. The sample size of 8 mice per group was based on the observed effect size in this model. No mice or samples were excluded from the analysis. The mice were euthanized on Day 15 by CO_2_ asphyxiation, following which the lungs were removed, rinsed in PBS and then fixed in Fekete’s solution prior to counting all lung tumour nodules.

### 2.8. IMQ-P Study

Imiquimod-induced psoriasis as a model is a simple and cheap way to emulate acute psoriasis and has been increasingly utilized to examine potential psoriasis therapies and better understand inflammation due to psoriasis. To determine the group size for this experiment, a power analysis was performed following a pilot study which determined that an n of 8 animals per group is sufficient to detect an approximately 19% difference between groups with 80% power. The experiment was conducted using female C57BL/6 mice (9–10 weeks old) that were randomized into vehicle control and peptide treatment groups (8 mice per group). All animal staff were aware of group allocation and dosing information. Mice were anaesthetized with 3% isofluorane and their backs shaved and depilated, followed by baseline scoring as per Table 1 below. On Day 0, i.e., prior to application of imiquimod (IMQ) cream, mice were injected intraperitoneally with either vehicle (sterile MilliQ water) or peptide IK14004 (MW = 2021 g/mol; 400 µg/200 µL MilliQ water). Starting on study Day 1, approximately 50 μL of 5% IMQ cream (Perrigo Inc., Allegan, MI, USA) was applied to the hair-free skin on the backs of mice and rubbed in until absorbed. The IMQ cream was applied daily for 4 days. Repeated injections of vehicle and peptide were administered on alternate days, i.e., Days 2 and 4 and on Day 5, i.e., one day after the last injection. Subjective Psoriasis Area and Severity Index (PASI) scores of dorsal skin were determined for each mouse based on erythema and scaling and the cumulative PASI score calculated. PASI scores were determined on Day 5 followed by euthanasia (3% Isoflurane with 1 L/min of oxygen) prior to exsanguination and cervical dislocation.

For histopathological analyses, approximately 2 × 2 cm samples of affected dorsum skin from all mice were fixed in 10% neutral buffered formalin. Skin samples were processed, sectioned (4 µm), and stained with Hematoxylin and Eosin (H & E) using standard methods. Histopathologic features evaluated included epidermal thickness, inflammation within the dermal and epidermal/keratin layers and epidermal erosions when present. The data and scores were entered into an appropriate Microsoft Office application.

The Histopathologic Scoring system developed by Inotiv Boulder, CO, USA, is designed to capture treatment effects as indicated in the Table 2 below. Scoring is based on evidence of epidermal hyperplasia, dermal inflammatory cell infiltrates, epidermal inflammatory cell accumulation including within the keratin layer and epidermal erosions/ulceration when present. Inflammatory cell infiltrates often extend into the underlying dermis and are occasionally intermixed with variable amounts of oedema.

### 2.9. Data Analyses

All data are expressed as the mean ± standard error of the mean (SEM). Flow cytometry data were exported as FCS files from Attune™ NxT software (version 2.0) and analysed using FlowJo software™ (v11) from which data were tabulated for export to Microsoft Excel. Statistical analyses for flow cytometry/ELISA assays were performed using GraphPad Prism (version 8.4.2) on a Windows Operating System. Data from in vitro studies were analysed by means of either one-way or two-way ANOVA with Dunnett’s post-test or Holm–Sidak’s post-test for peptide dose/vehicle comparisons. Single-dose compound effects were analysed by paired *t*-test. Data from the melanoma study were analysed in the GraphPad Prism version 7 using a one-way ANOVA followed by Dunnett’s post-hoc test. In the IMQ-induced psoriasis study, the cumulative score of the severity of disease (total = 0–10) was analysed by the non-parametric Mann–Whitney U test. Statistically significant differences between vehicle control and peptide-treated samples are indicated in the Figure panels as * *p* < 0.05, ** *p* < 0.01, *** *p* < 0.001 and **** *p* < 0.0001.

## 3. Results

### 3.1. IK14004 Suppresses Expression of IL-12Rβ2 in Isolated T Cell Cultures and Differentially Activates STAT3/STAT4

**Figure 1:**
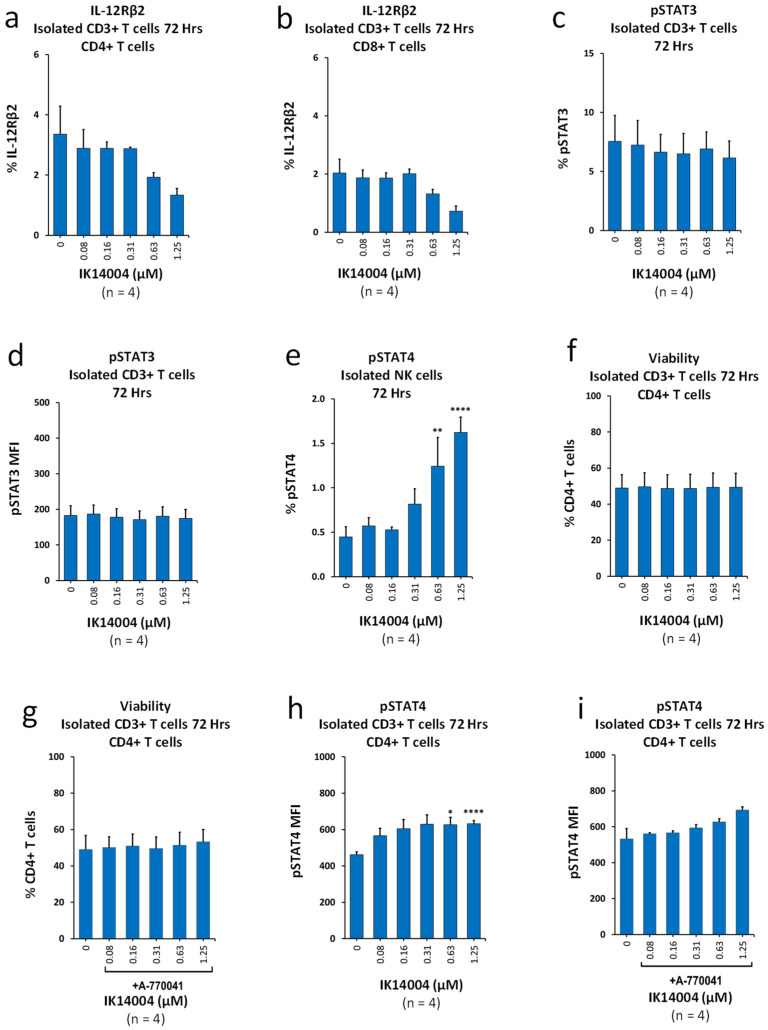
IK14004 suppresses expression of IL-12Rβ2 in isolated T cell cultures and differentially activates STAT3/STAT4. (**a**) Percentage of IL-12Rβ2-expressing CD4+ T cells within stimulated CD3+ T cultures. (**b**) Percentage of IL-12Rβ2-expressing CD8+ T cells within stimulated CD3+ T cultures. (**c**) Percentage of pSTAT3-expressing CD3+ T cells exposed to IK14004. (**d**) Expression level (MFI) of pSTAT3 in CD3+ T cells exposed to IK14004. (**e**) Percentage of pSTAT4-expressing NK cells within isolated NK cell cultures exposed to peptide. (**f**) Viability of stimulated CD3+ T cells exposed to peptide in the absence of Lck inhibitor (A-770041). (**g**) Viability of stimulated CD3+ T cells exposed to peptide in the presence of Lck inhibitor (A-770041). (**h**) Expression level (MFI) of pSTAT4 in CD4+ T cells within stimulated CD3+ T cell cultures exposed to peptide in the absence of Lck inhibitor (A-770041). (**i**) Expression level (MFI) of pSTAT4 in CD4+ T cells within stimulated CD3+ T cell cultures exposed to peptide in the presence of Lck inhibitor (A-770041). Dose-dependent trends seen in (**a**,**b**,**i**) were not statistically significant as assessed by one-way or two-way ANOVA with Dunnett’s post-test. The duration of experiments is indicated above each panel expressed as “Hrs”. The descriptor “n”; below each panel indicates the number of donor samples (experimental replicates). * *p* < 0.05, ** *p* < 0.01 and **** *p* < 0.0001.

Peptide IK14004 destabilises DCs with inhibition of IL-12 production [24]. We therefore sought to assess the effect of the peptide on expression of the β2 signalling chain in the IL-12p70 receptor complex in T cells in the absence of DC-T cell interactions. In contrast to peptide-enhanced expression of β2 in T cells in the presence of DCs [25], the proportion of IL-12Rβ2-expressing CD4+ T cells decreased (Figure 1a) as did the proportion of IL-12Rβ2-expressing CD8+ T cells (Figure 1b). To determine whether STAT3 plays a role in these events, we then examined the effect of IK14004 on activation of STAT3 in CD3+ T cell cultures, and neither the proportion of pSTAT3-expressiing T cells (Figure 1c) nor the expression level (MFI) (Figure 1d) was enhanced by the peptide. Given that IK14004 activates STAT4 in CD3+ T cells [25], we next assessed its effect on STAT4 activation in NK cells. Exposure of isolated NK cells to the peptide resulted in a 3-fold increase in the proportion of pSTAT4-expressing NK cells at the highest peptide concentration (Figure 1e).

Activation of the T cell receptor (TCR) is regulated by lymphocyte-specific protein tyrosine kinase (Lck) [43], and IL-2 can activate Lck [44]. In contrast to Lck-mediated psoriasis [45], STAT4 is not involved [46]. Given that both IL-2 and pSTAT4 are induced by IK14004 in TCR-stimulated CD4+ T cells [25], we examined the effect of Lck inhibition on peptide-mediated STAT4 activation in CD3+ T cells exposed to the Lck inhibitor, A-770041 [47]. Peptide alone did not affect the viability of CD4+ T cells within TCR-activated CD3+ T cell cultures (Figure 1f), and neither was viability affected by the combination of the peptide and A-770041 (100 nM) (Figure 1g). Furthermore, the dose-dependent increase in the expression level (MFI) of pSTAT4 in CD4+ T cells in the presence of the peptide alone (Figure 1h) was mirrored upon addition of the Lck inhibitor (Figure 1i) although this was not statistically significant. Supplementary data: Figure S2: Refers to manuscript Figure 1a,b, Figure S3: Refers to manuscript Figure 1c,d, Figure S4: Refers to manuscript Figure 1e, Figure S5: Refers to manuscript Figure 1f,g, Figure S6: Refers to manuscript Figure 1h,i. 

### 3.2. IK14004 Lowers the IFN-α:IFN-β Ratio in T Cells, but Not NK Cells, and Does Not Induce Production of IL-23 or Tumour Necrosis Factor-Alpha (TNF-α)

**Figure 2:**
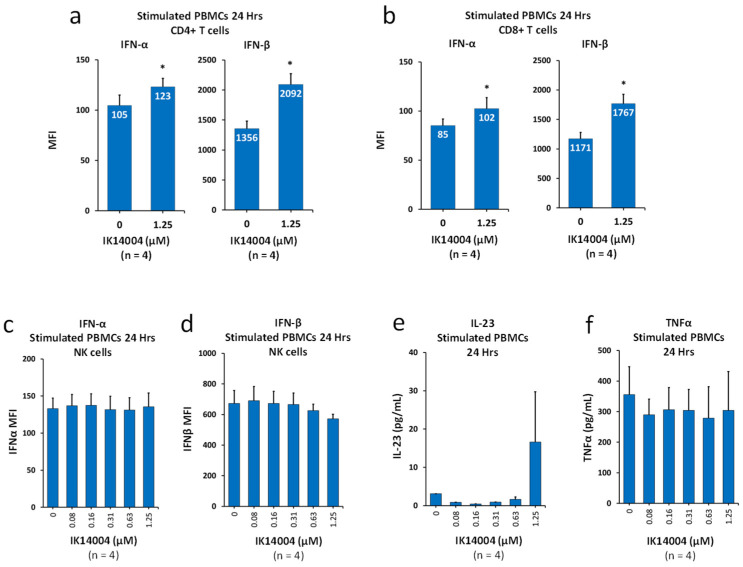
IK14004 lowers the IFN-α:IFN-β ratio in T cells, but not NK cells, and does not induce production of IL-23 or tumour necrosis factor-alpha (TNF-α). (**a**) Expression levels (MFI) of IFN-α and IFN-β in CD4+ T cells within stimulated PBMC cultures exposed to peptide (1.25 µM) showing percentage increases in IFN-α/β expression above basal levels. (**b**) Expression levels (MFI) of IFN-α and IFN-β in CD8+ T cells within stimulated PBMC cultures exposed to peptide (1.25 µM) showing percentage increases in IFN-α/β expression above basal levels. (**c**) Expression levels (MFI) of IFN-α in NK cells within stimulated PBMC cultures. (**d**) Expression levels (MFI) of IFN-β in NK cells within stimulated PBMC cultures. (**e**) IL-23 levels in supernatant from stimulated PBMC cultures exposed to IK14004. (**f**) TNF-α levels in supernatant from stimulated PBMC cultures exposed to IK14004. IL-23 levels (**e**) were below detection levels of the kit, and the reported values were extrapolated. Hence, statistical tests were not applied, and there was no statistically significant effect of the peptide on either IFN-β production by NK cells (**d**) or TNF-α production (**f**) as assessed by one-way ANOVA with Dunnett’s post-test. The duration of experiments is indicated above each panel expressed as “Hrs”. The descriptor “n”; below each panel indicates the number of donor samples (experimental replicates). * *p* < 0.05.

The beneficial effects of a low IFN-α:IFN-β ratio in suppressing autoimmune diseases including psoriasis are widely recognised [26,27,28,29,30,31,32,33]. Although IK14004 induces expression of Type I IFNs in T cells [25], whether the IFN-α:IFN-β ratio is altered has never been reported. We therefore compared the increase in intracellular expression of IFN-α when exposed to peptide at a concentration of 1.25 µM with the increase in IFN-β. As indicated by the MFI values within histogram bars, in CD4+ T cells, the IFN-α:IFN-β ratio in the presence of vehicle control was reduced from 1:12.9 to 1:17.0 in the presence of IK14004 (Figure 2a). Similarly, in CD8+ T cells, the IFN-α:IFN-β ratio in the presence of vehicle control was reduced from 1:13.8 to 1:17.3 in the presence of IK14004 (Figure 2b). We next assessed the effect of peptide on expression of IFN-α/β in NK cells within TCR-activated PBMC cultures. Expression levels of IFN-α remained unaltered in the presence of IK14004 (Figure 2c), and a non-significant decrease in IFN-β expression was observed at higher doses (Figure 2d). Given that IL-23 and TNF-α are also implicated in the pathogenesis of psoriasis in murine models [45], we then examined the effect of IK14004 on production of these cytokines in PBMC cultures. Neither expression of IL-23 (Figure 2e) nor TNF-α (Figure 2f) was significantly altered in the presence of peptide. Supplementary data: Figure S7: Refers to manuscript Figure 2a,b, Figure S8: Refers to manuscript Figure 2c,d. 

### 3.3. IK14004 Differentially Affects Expression of IL-15 and IL-2 Receptors in CD3+ T and NK Cells

**Figure 3:**
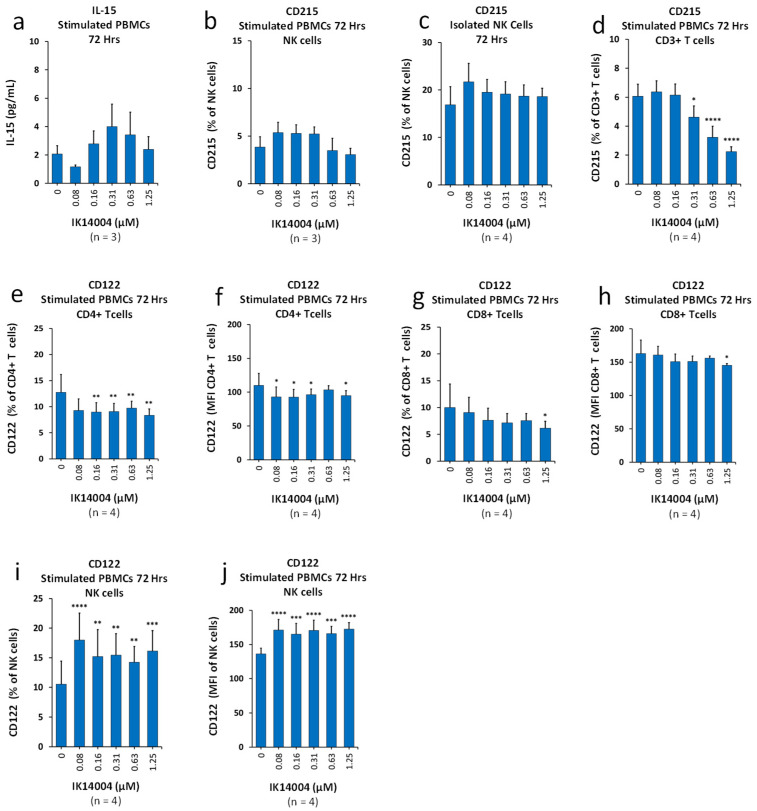
IK14004 differentially affects expression of IL-15 and IL-2 receptors in CD3+ T and NK cells. (**a**) IL-15 levels in supernatant from stimulated PBMC cultures. (**b**) Percentage of CD215 (IL-15α)-expressing NK cells within stimulated PBMC cultures. (**c**) Percentage of CD215 (IL-15α)-expressing cells in isolated NK cell cultures. (**d**) Percentage of CD215 (IL-15α)-expressing CD3+ T cells within stimulated PBMC cell cultures. (**e**) Percentage of CD122 (IL-2Rβ)-expressing CD4+ T cells within stimulated PBMC cell cultures. (**f**) Expression levels (MFI) of CD122 in CD4+ T cells within stimulated PBMC cell cultures. (**g**) Percentage of CD122 (IL-2Rβ)-expressing CD8+ T cells within stimulated PBMC cell cultures. (**h**) Expression levels (MFI) of CD122 in CD8+ T cells within stimulated PBMC cell cultures. (**i**) Percentage of CD122 (IL-2Rβ)-expressing NK cells within stimulated PBMC cell cultures. (**j**) Expression levels (MFI) of CD122 in NK cells within stimulated PBMC cell cultures. The duration of experiments is indicated above each panel expressed as “Hrs”. The descriptor “n”; below each panel indicates the number of donor samples (experimental replicates). * *p* < 0.05, ** *p* < 0.01, *** *p* < 0.001 and **** *p* < 0.0001.

The co-expression of the three IL-2 receptor subunits, IL-2Rα (CD25), IL-2Rβ (CD122) and a γ subunit (γ_c_ or CD132), is needed to confer high-affinity binding of IL-2 to a cell [48], and IL-15 also binds to CD122 [49]. We first examined the effect of the peptide on IL-15 secretion in PBMC cultures, and IK14004 did not induce a dose-dependent effect on the production of IL-15 (Figure 3a). IL-15 binds to the high-affinity IL-15α (CD215) subunit for presentation in *trans* to neighbouring cells [15], and we then compared the effect of peptide on the expression of CD215 in NK versus CD3+ T cells. Exposure of cells to the peptide did not affect the proportion of CD215-expressing NK cells either within TCR-stimulated PBMC cultures (Figure 3b) or in isolated NK cell cultures (Figure 3c). In contrast, a marked reduction in CD215-expressing CD3+ T cells within TCR-activated PBMC cultures was observed in the presence of peptide (Figure 3d). We next sought to determine the effect of IK14004 on CD122 expression in immune cell subsets within TCR-stimulated PBMC cultures. Exposure to the peptide reduced the proportion of CD122-expressing CD4+ T cells (Figure 3e) and level of expression (MFI) (Figure 3f). Similarly, exposure to the peptide reduced the proportion of CD122-expressing CD8+ T cells (Figure 3g) and level of expression (MFI) (Figure 3h). In contrast, IK14004 at the lowest dose (0.08 uM) enhanced the proportion of CD122-expressing NK cells (Figure 3i) and level of expression (MFI) (Figure 3j). Supplementary data: Figure S9: Refers to manuscript Figure 3b, Figure S10: Refers to manuscript Figure 3c, Figure S11: Refers to manuscript Figure 3d, Figure S12: Refers to manuscript Figure 3e–h, Figure S13: Refers to manuscript Figure 3i,j. 

### 3.4. IK14004 Inhibits Melanoma Growth and Psoriasis in Murine Models

**Figure 4:**
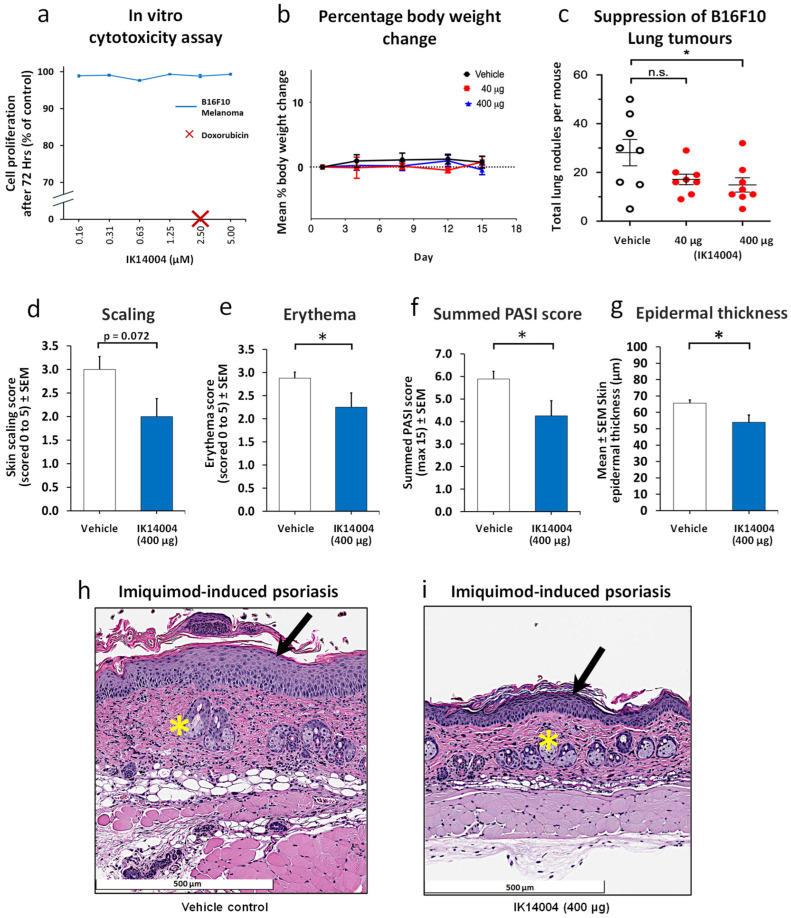
IK14004 inhibits melanoma growth and psoriasis in murine models. (**a**) Proliferation of B16F10 melanoma cells in vitro in the presence of IK14004 and Doxorubicin after 72 h. (**b**) Mean % body weight changes in vehicle control and peptide-treated mice. (**c**) B16F10 lung tumours after 2 weeks of treatment with IK14004 (4 doses). (**d**) Peptide-mediated inhibition of scaling in the Imiquimod-induced psoriasis (IMQ-P) model. (**e**) Peptide-mediated inhibition of erythema in the IMQ-P model. (**f**) Peptide-mediated inhibition of the summed Psoriasis Area and Severity Index (PASI). (**g**) Peptide-mediated reduction in epidermal thickness in the IMQ-P model measured microscopically. (**h**) H & E-stained skin section from a vehicle-treated mouse in the IMQ-P model showing epidermal hyperplasia (black arrow) and dermal inflammatory infiltrates (yellow asterisk). (**i**) H & E-stained skin section from an IK14004-treated mouse in the IMQ-P model showing decreased epidermal hyperplasia (black arrow) and reduced dermal inflammatory infiltrates (yellow asterisk). * *p* < 0.05 and n.s. indicates not significant.

Given the known link between melanoma and autoimmune comorbidities [1] we chose to investigate the effect of IK14004 at two doses on melanoma growth in vivo. Previously reported pharmacokinetic studies have shown that retention of IK14004 in the blood after 24 h approximates 0.75 µM which is within the effective dose range of the peptide (0.08–1.25 µM) when tested on human immune cells in vitro [25]. In preliminary experiments, in vitro cultures of B16F10 melanoma cells were exposed to IK14004 for 72 h, and no cytotoxic effects were observed at 5 µM in contrast to total cell kill by Doxorubicin at a concentration of 2.5 µM (Figure 4a). In the melanoma tumour model, we selected the same dose regimen of IK14004 (400 µg administered intraperitoneally twice weekly for two weeks) that has been reported to inhibit Lewis lung cancer (LLC) in both tumour allograft and liver metastasis models [25] and included a lower dose (40 µg). Lack of toxicity prior to euthanasia on Day 15 at the higher dose was confirmed by finding no change in mean percentage body weights compared with vehicle control-treated mice (Figure 4b). At the higher dose (400 µg), IK14004 significantly inhibited B16F10 tumour development in the lung, and a non-significant trend in growth inhibition was observed at the lower dose (Figure 4c).

Exacerbation of pre-existing psoriasis or eruption of de novo disease is an established side effect of anti-PD-1 therapy for various cancers including melanoma [50,51,52]. We therefore chose to investigate the effect of the peptide in a psoriasis model using the higher dose tested in the melanoma study. The first peptide dose was administered prior to the first application of IMQ cream as has previously been reported for the evaluation of anti-PD-1 in this model [53]. At the termination of the study, peptide-treated mice exhibited less scaling (Figure 4d) and erythema (Figure 4e) than vehicle-treated control animals including a lower summed PASI score (Figure 4f). Histopathological analyses were conducted after euthanasia as described in the Methods, and measurement of epidermal thickness in skin sections revealed a significant reduction in epidermal thickness in peptide-treated mice (Figure 4g). Epidermal hyperplasia (black arrow) and dermal inflammatory cell infiltrates (yellow asterisk) were apparent on H & E-stained skin sections in the absence of peptide (Figure 4h). In peptide-treated mice, epidermal hyperplasia and dermal inflammatory infiltrates were reduced (Figure 4i).

## 4. Discussion

A range of autoimmune diseases that include psoriasis are positively correlated with metastatic melanoma [53]. Although anti-PD-1 cancer therapy is effective against metastatic melanoma [3,36], it exacerbates psoriasis in patients [51] and in murine models [54]. Garris and colleagues [36] have suggested that successful anti-PD-1 therapy depends on the positive feedback loop between IL-12p40 and IFN-γ consequent upon T cell/DC interactions. In support of this notion, we have previously confirmed that anti-PD-1 mAb enhances production of these two cytokines by human PBMCs when tested in vitro [25]. However, peptide IK14004 exerts opposite effects to anti-PD-1 and inhibits IL-12p40/IFN-γ production [25]. Response rates to anti-PD-1 therapy in non-small cell lung cancer patients are less than 25% [55] and minimal in anti-PD-1-treated mice bearing Lewis lung cancer (LLC) tumours [56]. In contrast, IK14004 significantly inhibits in vivo growth of LLC in two murine models [25]. In the present report, we provide further evidence from human immune cells tested in vitro and murine melanoma/psoriasis models in support of the hypothesis that it may be possible to reconcile effective cancer immunotherapy with preservation of immune tolerance.

Previous pharmacokinetic studies following intravenous administration have indicated retention of IK14004 in the heart (a surrogate for blood levels) after several hours [25]. In the melanoma and psoriasis studies reported herein, the peptide was administered intraperitoneally (IP). We have also reported that biodistribution and clearance following IP administration of radiolabelled peptide indicates persistence of IK14004 in the circulation at 24 h post administration as well as accumulation in the spleen over the same time frame [25]. This is congruent with an aqueous soluble peptide capable of distributing throughout the body following IP administration with maintenance in the circulation at biologically relevant levels over hours to days.

The IK14004 compound is comprised of a short amino acid sequence, RSKAKNPLYR, linked to branched dodecanoic (lauric) acid residues. The branched lipid unit is thought to enhance membrane penetration and protect the peptide from enzymatic attack within cells [24]. Although we have not established where within human immune cells the peptide exerts its effects, a FITC-labelled multimer of the peptide minus the lipid component rapidly enters the nucleus of cancer cells [57]. Moreover, within the 10-mer is an RSKAK motif which could play a role in gene induction given that a homologous stretch of amino acids within the nuclear localisation sequence of tumour inhibitor of growth 4 (ING4) protein, i.e., RARSK, binds to p53 located in the nucleus [58].

An integrated understanding of how signal transducers and activators of transcription combine to maintain anticancer cell immunity without compromising immune tolerance is lacking. STAT3 establishes a reciprocal relationship between melanoma cells and immune cells in favour of tumour evasion [53] and plays an important role in regulating the psoriasis-inducing IL-23/IL-17 axis [42]. For example, in the imiquimod (IMQ)-induced psoriasis model, enhanced features of psoriatic inflammation are associated not only with increased expression of STAT3, NF-kB, IFN-γ and TNF-α in CD4+ T cells but also activation of lymphocyte-specific protein tyrosine kinase (Lck) [45]. In contrast, STAT4 does not appear to be the culprit gene promoting autoimmune responses in murine models [46,59]. Whether this is related to the specific interaction required between the IL-12 receptor β2 subunit (IL-12Rβ2) and STAT4 for DNA binding and transcriptional activity of STAT4 remains to be determined, but it does not occur with STAT3 [60]. We have previously reported increased phosphorylation of STAT4 in CD4+ T cells exposed to either recombinant IL-12p70 or IK14004 [25]. In the present study we demonstrate that IK14004 induces a decreasing, albeit not significant, trend in expression of STAT3 in human CD4+ T cells and does not alter secretion of TNF-α.

Interestingly, chemotherapy drugs promote the ubiquitination and proteasomal degradation of STAT4 [61], and STAT4 has been associated with favourable prognoses in several cancer types [62]. Moreover, activation of antigen-presenting cells in the murine B16F10 melanoma model stimulates the IL-12/STAT4 signalling pathway [63]. To add to this complexity, IL-12 elevates the responses of activated human CD4+ T cells for further TCR stimulation by altering signalling pathways that include activation of Lck [43]. An immunologically suppressed state involving T cell function in melanoma is well-recognised, and it has been proposed that Lck inhibits immune escape of melanoma cells in vivo by promoting activation of T cells [64].

In contrast to melanoma, inhibition of Lck has been proposed to counteract psoriatic inflammation based on amelioration of clinical features, attenuation of Th17 immune responses and upregulation of Tregs in IMQ-treated mice treated with the Lck inhibitor, A-770041 [45]. While a selective approach to inhibition of Lck in tumour cells may be appropriate, suppression of anticancer responses by Lck-bearing, tumour-infiltrating lymphocytes needs to be avoided [65]. Furthermore, given involvement of the Lck/NF-kB signalling pathway in cancer cell signalling and in T cell function, blocking over-activation of NF-kB by inhibiting Lck has been suggested for the treatment of both inflammatory disorders and malignancies [66]. In the context of melanoma and psoriasis, a better understanding of how Lck, IL-12p70 and STAT4 regulate signalling pathways in these divergent pathologies appears warranted. Notwithstanding that IK14004 has been shown to bind to Lck in an ELISA [24], in the present study we established that IK14004-mediated activation of STAT4 in a human CD4+ T cell is not dependent on activation of Lck.

As for Lck, IL-2 sits at the crossroads of tolerance versus activation [67]. For example, the efficacy of low-dose IL-2 in the treatment of psoriasis patients has recently been confirmed [20], and clinical trials are currently under way using low-dose IL-2 in patients with rheumatoid arthritis, systemic lupus erythematosus, ankylosing spondylitis, Behcet’s syndrome, Sjogren’s syndrome and autoimmune hepatitis [68]. On the other hand, NK cell cytolytic function is severely impaired in STAT4 knockout mice [69], and IL-2 primes NK cells to be more responsive to IL-12 signalling through STAT4 activation [70]. This is thought to be mediated through enhanced expression of the IL-12p70 receptor chain, IL-12Rβ2 [71]. Peptide IK14004 increases expression of IL-12Rβ2 in NK cells including expression of NK cell-activating receptors, NKG2D/NKp44, in isolated NK cell cultures in the absence of IL-2 induction [25]. Given our finding in the current study that IK14004 promotes activation of STAT4 in isolated NK cells (Figure 1), we speculate that it may act as an IL-2-like “influence factor”, and whether the peptide plus recombinant IL-2 can exert an additive, or even synergistic effect, on the activation of NK cells warrants further investigation. Furthermore, activation of NK cells in an IL-2-independent manner may allow for available IL-2 that is induced by the peptide to bind to Tregs.

Type I IFNs also exhibit dual roles in the maintenance of immune tolerance and anticancer immunity. Antitumour activities include direct lymphocyte-mediated tumour cell killing, and IFN-α/β have been approved for the treatment of multiple malignancies [72]. However, IFN-α downregulates IL-2 with a negative effect on immunosuppressive Tregs which can be restored by addition of IL-2 [73]. In contrast, IFN-β induces proliferation of immunosuppressive Tregs [29]. Hence, a mechanistic connection exists between the immunosuppressive effects of IFN-β and Treg cells which has therapeutic implications for autoimmunity and malignancy [30].

Importantly, IFN-γ drives Treg cell fragility [74] which together with IL-12p40-mediated suppression of Treg cell function [11] promotes antitumour immunity. The lower IFN-α:IFN-β ratio induced by the peptide secondary to the greater expression of IFN-β in T cells (Figure 2) not only favours immune tolerance [26,27,28,29,30,31,32,33] but also suggests a possible mechanism underlying IK14004-mediated suppression of IFN-γ production by T cells [24]. For example, IFN-β inhibits IL-12p40 secretion by isolated mature DCs [75] as does IK14004 [24], and whether peptide-mediated induction of IFN-β expression in T cells indirectly regulates IL-12p40 production by DCs remains to be tested. Notably, IFN-β inhibits IL-12-mediated IFN-γ production via the canonical STAT4 signalling pathway activated upon DC-T cell interactions [76]. Both IL-12p40 and IFN-γ play a role in exacerbating psoriasis [37,38,40] in contrast to IFN-β [32] which also suppresses growth of melanoma more effectively than IFN-α [35]. Interestingly, peptide-mediated induction of IFN-β expression in T cells was not mirrored in NK cells suggesting that IK14004-induced activation of STAT4 is unlikely to be regulated via Type I IFN-mediated signalling events in NK cells.

It is also relevant that improvement in some patients with multiple sclerosis treated with IFN-β is thought to be underpinned by upregulation of the signalling receptor subunit for IL-12p70, i.e., IL-12Rβ2 [77]. In the present study we examined the effect of the peptide on the β2 signalling chain in the IL-12 receptor complex expressed in T cells. Interestingly, a dose-dependent decline in the proportion of IL-12Rβ2-expressing CD4+/CD8+ T cells was observed in the presence of IK14004. We suggest this reflects internalisation of the IL-12 receptor complex when bound to available IL-12p70. Taken together with peptide-induced IL-12p70 production by T cells [24] and activation of STAT4 by either recombinant IL-12p70 or IK14004 [25], we propose a novel signalling paradigm in T cells that involves IL-12p70-mediated activation of T cells in an autocrine manner in the absence of DC contact. Importantly, IL-12p40 is known to suppress responsiveness to IL-12p70 by competitively inhibiting IL-12p70 binding to the β1 chain of the IL-12Rβ1/Rβ2 receptor complex in both mouse and human systems [78]. Hence, the destabilising effect of IK14004 on isolated monocyte-derived DCs associated with suppression of IL-12p40 production [24] might serve to support such an autocrine signalling mechanism in T cells.

A paradox exists in immune checkpoint inhibitor (ICI) therapy in so far as IFN-γ and IL-12p40 appear critical for the preservation of cancer immunity in the presence of anti-PD-1 therapy on the one hand [36], while on the other hand autoimmune pathologies such as psoriasis [37,38,40] and systemic lupus erythematosus [39] are linked to the presence of these cytokines. The exacerbating effect of IL-12p40, as opposed to IL-12p70, in psoriasis is highlighted by improvement in psoriasis patients treated with a neutralising antibody against IL-12p40 [38]. In contrast, inhibition of the IL-12p70 isoform or IL-12Rβ2 knockout in murine psoriatic models worsens the disease [79]. Hence, the efficacy of Tofacitinib in psoriatic patients is thought to be related, at least in part, to suppression of the IL-12p40 subunit in DCs upon activation by IFN-γ signalling [80].

The IL-12 isoform, IL-12p70, can control autoimmune inflammation [12], and a neutralising antibody against IL-12p40 is beneficial in psoriatic patients [38]. Interestingly, Th1-like immunosuppressive Tregs depend on IL-12p70 and IFN-γ for their generation [81], yet enhanced IFN-γ expression in CD4+ T cells characterises the murine psoriasis model [45]. Taken together with opposing effects of IL-12p70 versus IL-12p40 on Treg cell function [11,12], it is not surprising that IL-12p40/IFN-γ mediated signalling in anti-PD-1 cancer therapy [36] is linked to exacerbation of psoriasis [50,51,52]. We have previously reported the peptide-mediated induction of activating receptors (NKG2D/NKp44) in NK cells [25] and suggest that, in combination with increased numbers of pSTAT4-expressing NK cells in the presence of IK14004, this may compensate for the peptide’s suppressive effect on the IL-12p40/IFN-γ feedback loop considered responsible for anti-PD-1 efficacy in cancer patients [36]. For example, the human perforin gene is a direct target of STAT4 activated by IL-12 in NK cells [82]. Furthermore, inhibition of IFN-γ production by peptide is relevant because IFN-γ negatively regulates expression of NKG2D [83]. Notably, melanoma cells hinder the immune function of NK cells by suppressing their expression of NKG2D and NKp44 [84], and given that IL-2 is considered necessary to render NK cells functionally competent upon NKG2D engagement [85], the IL-2-like effect of IK14004 on NK cells could be helpful.

Another potential benefit of inhibiting IFN-γ production is that IFN-γ-expressing Tregs lose suppressive capacity and may promote autoimmune disease in humans [86]. Tregs are a heterogeneous population comprised of multiple cell states that play a pivotal role in maintaining immune tolerance although they also have a detrimental impact in the tumour microenvironment by preventing antitumour responses [87]. Th2-like Tregs secrete higher levels of IL-4 than IL-2 which supports a tumorigenic environment [87]. Given that IK14004 suppresses IL-4 while enhancing IL-2 production by T cells [24], we believe that peptide-mediated expansion of Tregs [24] reflects a Th1-like Treg phenotype although this has not been definitively established. In autoimmune disease, inflamed tissues have high T effector (Teff)-to-Treg ratios, and Th1-like Tregs, that do not express IFN-γ, are adapted to suppress Teff cells [86]. Hence, this serves to limit Th1-mediated inflammation [86] and be of benefit in autoimmune conditions such as psoriasis [88] where Tregs are considered dysfunctional [89]. On the other hand, the impact of IK14004 on interactions between an expanded Th1-like Treg population and DCs in a tumour microenvironment [81] together with induction of a more immature DC phenotype [24] would appear to be disadvantageous to antitumour immunity [90].

Redirecting IL-15 towards NK cells, as opposed to T cells (Figure 3), may serve to maintain functionality of both Tregs and NK cells and thus minimise autoimmune responses in the presence of enhanced cancer surveillance by the immune system. IL-15 renders T cells resistant to the suppressive function of conventional Tregs, thereby, maintaining IFN-γ production by T cells [91]. Hence, in the presence of IL-15, IFN-γ production by CD4+/CD8+ T cells cannot be efficiently inhibited by Tregs [91]. In addition, IL-15 is the most potent soluble mediator enabling NK cell maturation [15], and IL-15 receptor signals are critical for NK cell development [17]. NK cells express the CD122 (IL-2Rβ)/γ_c_ receptor chains and are activated in *trans* by cells presenting IL-15 bound to IL-15a (CD215) [92]. Importantly, IL-15 shares binding with IL-2 to the IL-2 receptor component, CD122 [93], which has been elegantly demonstrated by the complete inhibition of IL-15-enhanced NK cytotoxicity in the presence of anti-IL-2Rβ monoclonal antibody [49].

Whether the low levels of IL-15 seen in the present study in the absence/presence of peptide reflect IL-15 bound to its high affinity receptor subunit, IL-15α, or decreased production of IL-15 by less mature DCs was not established. However, the significant decrease in IL-15α-expressing CD3+ T cells in the presence of a peptide combined with opposing effects on CD122 expression in T and NK cells suggests a mechanism whereby Treg functionality could be retained in the presence of NK cell activation by IL-15. Furthermore, NKG2D signalling is coupled to the IL-15 receptor pathway [94] which has relevance to melanoma. For example, IFN-γ downregulates NKG2D ligand expression in melanoma cells which impairs NKG2D-mediated cytolysis of MHC class I-deficient melanoma by NK cells [95], and IL-15 can partially overcome this tumour escape mechanism [96].

To the best of our knowledge, IK14004 is the first example of a peptide exerting inhibitory effects in murine models of both cancer and an autoimmune disease at the same dose. Pharmacokinetic data have shown that peptide concentrations achievable in vivo that result in suppression of Lewis lung cancer (LLC) growth in metastases and subcutaneous allograft models do not inhibit proliferation of LLC cells in vitro [25]. Similarly, in the present study, IK14004 does not inhibit proliferation of B16F10 melanoma cells in vitro after 72 h at concentrations much higher than achievable in vivo which supports an immunomodulatory effect as suggested by findings in human immune cell subsets [24,25]. However, a limitation of our study is that we did not seek to determine the immunological mechanisms underlying peptide-mediated suppression of B16F10 lung tumours. The greater induction of IFN-β than IFN-α as seen in human cells could be relevant here given that IFN-β exerts antitumour effects against melanoma and generally is more potent than IFN-α [35]. Importantly, species differences in the immune systems between mice and humans also need to be considered. For example, unlike human NK cells, naïve mouse NK cells are devoid of perforin and granzyme B cytotoxic granules [15]. In contrast, production of perforin and granzymes is a feature of CD56^dim^ NK cells which comprise more than 90% of human peripheral blood NK cells [97]. Moreover, the complete absence of the natural cytotoxic receptor, NKp44, in mice [98] and lack of expression of NKG2D ligand in B16F10 melanoma cells [99] indicate that IK14004-mediated activation of NK cells via expression of NKp44/NKG2D, as shown in human cells [25], is unlikely to account for the suppression of melanoma lung tumours by peptide. Nevertheless, we cannot discount the possibility that IK14004 may counter a tumour escape mechanism arising from expression of the IL-12 receptor chain, IL-12Rβ2, in murine melanoma cells. This is thought to divert available IL-12p70 away from neighbouring immune cells [100], and IK14004-mediated induction of the IL-12 receptor complex in murine NK cells [25] may compete with this process. Further testing of additional tumour types in pre-clinical animal models is warranted, and if IK14004 were to suppress advanced melanoma in patients, then the mechanism would necessarily differ from that considered responsible for effective anti-PD-1 therapy [36].

We also acknowledge several limitations of our study relevant to the psoriasis model. Firstly, we did not examine the effect of peptide on psoriasis exacerbated by either PD-1 knockout or anti-PD-1 antibody in IMQ-treated mice [53]. Secondly, we did not study the animal model of psoriasis in more detail to determine effects on the cellular infiltrate in the skin. For example, we did not assess the presence of neutrophils, macrophages or CD8+ T cells and the effect of peptide on DC maturation and induction of Tregs was not determined. Thirdly, the effect of IK14004 on the expression of relevant cytokines such as the IL-12 isoforms, Type I IFNs, IFN-γ, IL-2, IL-23 and IL-17 was not analysed. Aldara (5% imiquimod)-induced acute skin inflammation has become the most widely used animal model of psoriasis during the past 15 years [101], and 5% IMQ cream was used in the present study. However, combining 5% IMQ cream plus anti-PD-1 mAb fails to reveal psoriasis-enhancing effects of this checkpoint blocker, and lower IMQ concentrations, such as 3.5%, are required [53]. Hence, studies in mice exposed to lower IMQ concentrations to determine whether IK14004 can lessen anti-PD-1 antibody-mediated exacerbation of psoriasis-like skin changes would be informative. In such studies, the inflammatory cell infiltrate, DC/T cell phenotypes, Tregs and relevant cytokines could be compared between mice exposed to either peptide or anti-PD-1 antibody alone and in mice treated with both compounds. In addition, examination of non-lesional and lesional skin explants obtained from psoriasis patients that have been exposed to IK14004 ex vivo is warranted. Importantly, psoriasis is a heterogeneous disease, and testing in different preclinical models is necessary because no single model can be expected to include all the pathogenic mechanisms underlying this disease in affected humans [102]. Moreover, to establish a convincing role for this peptide in both immunosuppressive and immuno-promoting activities will require further testing in other autoimmune and cancer models.

Emerging anticancer therapeutics include hybrid oncolytic peptides that target nuclear DNA in cancer cells [103,104,105]. Notably, cancer cell toxicity induced by these compounds in vitro occurs at relatively high micromolar concentrations [104]. In contrast, IK14004 elicits divergent stimulatory and inhibitory cytokine and cellular responses in vitro at nanomolar concentrations without direct effect on cancer cells [24,25]. In addition to oncolytic peptides, novel peptide drugs have emerged with potential to monitor expression of checkpoint inhibitor molecules, e.g., PD-L1, on cancer cells during cancer immunotherapy [106]. Importantly, IFN-γ is linked to expression of PD-L1 on cancer cells [107], and IK14004-mediated suppression of IFN-γ production by T cells in the absence/presence of DCs [24] raises the possibility that IK14004 may also regulate PD-L1 expression on cancer cells.

Our findings raise several questions. For example, why does IK14004-mediated expansion of Tregs not inhibit NK cell-activating responses? Tregs attenuate the action of IFN-y-secreting Teffs but do not inhibit IL-2-mediated activation of NK cells [108]. Hence, any possible additive or synergistic effects between IL-2 and peptide on NK cell activation would not compromise Treg cell functionality. Furthermore, given the contrasting effects of low dose IL-2 and IFN-γ on pathogenesis of autoimmune diseases, could induction of IL-2 and suppression of IFN-γ production by T cells be achieved simultaneously? Targeting the calcium/calmodulin-dependent protein kinase IV (CaMKIV) pathway may be one option to achieve this outcome. Yong and colleagues [109] have shown that CaMKIV expression is significantly increased in psoriatic lesional skin from psoriasis patients compared with healthy skin and that CaMKIV-deficient mice treated with imiquimod exhibit reduced severity of psoriasis compared with wild-type mice [109]. A potential role for CaMKIV-mediated modulation of IL-2 and IFN-γ in this condition cannot be ignored given that CaMKIV-mediated downstream signalling inhibits IL-2 transcription [109] while inducing IFN-γ production [110]. In this way, activation of CaMKIV-mediated signalling may compromise immune tolerance, and we have shown that IK14004 inhibits CaMKIV activity in non-cell-based kinase profiling assays [24].

It also seems reasonable to ask whether compounds can be developed that can act against contrasting diseases such as autoimmunity and cancer? Since deregulation of immunity is the main culprit in melanoma and autoimmune disease, the development of “multipotent drugs” that have potential to act against such divergent pathologies remains a challenge [54]. Autoimmunity and cancer are two sides of the same coin with the same molecular players working in opposite directions [111]. Hence, a caveat is that drugs which target both conditions may not completely inhibit both autoimmune responses and cancer in the same animal model or patient, and outcomes will remain contextual. For example, peptide-mediated activation of NK cells may be more relevant to targeting cells with a cancer stem cell (CSC) phenotype where targeting is mediated primarily via NKG2D-ligand interactions [112].

## 5. Conclusions

Our findings highlight the possibility that enhanced anticancer immunity can co-exist with immune tolerance. The peptide uncouples Type I IFN/IL-12p70/IL-2 signalling axes from IL-12p40-/IFN-γ-mediated signalling pathways. In combination with increased Tregs, suppressed DC maturation, activation of NK cells and inhibition of both cancer and psoriasis in mice, these data suggest that signalling mechanisms in cancer immunotherapy exist which could be exploited to minimise the occurrence of irAEs. Administration of a peptide combined with ICIs may also offer an opportunity to further evaluate these mechanisms in situations where cancer immunotherapy is likely to exacerbate pre-existing autoimmune disease.

## Figures and Tables

**Table 1:** In vivo assessment of the Psoriasis Area and Severity Index (PASI).

	0	1	2	3	4	5
**Erythema**	None	Faint Pink	Definite Pink	Pinkish-Reddish	Definite Red	Redness with Bleeding
**Scaling**	None	1–20%	21–40%	41–60%	61–80%	≥81%

**Table 2:** Ex vivo assessment of epidermal thickness (hyperplasia).

0	Normal, no hyperplasia, mean measure is ≤30 µm
0.5	Very minimal epidermal hyperplasia, mean measure is 31–45 µm
1	Minimal epidermal hyperplasia, mean measure is 46–60 µm
2	Mild epidermal hyperplasia, mean measure is 61–75 µm
3	Moderate epidermal hyperplasia, mean measure is 76–90 µm
4	Marked epidermal hyperplasia, mean measure is 91–115 µm
5	Severe epidermal hyperplasia, mean measure is >115 µm

Epidermal thickness in microns was determined by microscopic examination of post-mortem skin sections. Epidermal hyperplasia was estimated based on the thickness of viable epidermal cells, excluding the keratin layer. The mean of 4 epidermal thickness measurements at approximately equidistant sites across the entire length of the sample were recorded and scored.

## Data Availability

Correspondence and requests for materials should be addressed to M.A.

## Supplementary Materials

The following supporting information can be downloaded at: https://www.mdpi.com/article/10.3390/biomedicines13081908/s1, Figure S1: Refers to the structure, purity and amino acid analysis of IK14004, Figure S2: Refers to manuscript Figure 1a,b, Figure S3: Refers to manuscript Figure 1c,d, Figure S4: Refers to manuscript Figure 1e, Figure S5: Refers to manuscript Figure 1f,g, Figure S6: Refers to manuscript Figure 1h,i, Figure S7: Refers to manuscript Figure 2a,b, Figure S8: Refers to manuscript Figure 2c,d, Figure S9: Refers to manuscript Figure 3b, Figure S10: Refers to manuscript Figure 3c, Figure S11: Refers to manuscript Figure 3d, Figure S12: Refers to manuscript Figure 3e–h, Figure S13: Refers to manuscript Figure 3i,j.
