# Supplementary material for: An Immunomodulating Peptide with Potential to Promote Anticancer Immunity Without Compromising Immune Tolerance
Source: Biomedicines. 2025 Aug 5;13(8):1908. doi: 10.3390/biomedicines13081908 (PMC12383534; doi:10.3390/biomedicines13081908)
Supplement: Supplementary file 1 [file biomedicines-13-01908-s001.zip › biomedicines-3755516-supplementary.pdf]

An immunomodulating peptide with potential to promote anticancer immunity without compromising immune tolerance

Michael Agrez[1]*, Christopher Chandler[2], Amanda L. Johnson[3], Marlena Sorensen[3], Kirstin Cho[3], Stephen Parker[1], Benjamin Blyth[4], Darryl Turner[5], Justyna Rzepecka[5], Gavin Knox[5], Anastasia Nika[5], Andrew M. Hall[5], Hayley Gooding[5] and Laura Gallagher[5]

[1]InterK Peptide Therapeutics Limited, Sydney, New South Wales, Australia. [2]Auspep Pty Limited, Melbourne, Australia. [3]Inotiv Inc., Boulder, Colorado, USA. [4]Peter MacCallum Cancer Centre and Sir Peter MacCallum Department of Oncology, University of Melbourne, Melbourne, Australia. [5]Concept Life Sciences, Edinburgh, Scotland.

Figure S1: Refers to the structure, purity and amino acid analysis of IK14004

Sequence: H-Arg-Ser-Lys-Ala-Lys-Asn-Pro-Leu-Tyr-Arg-(2)Adod-(2)Adod-(2)Adod-(2)Adod-amide, where (2)Adod = (S)-2-aminododecanoic acid.

Structure of IK14004:

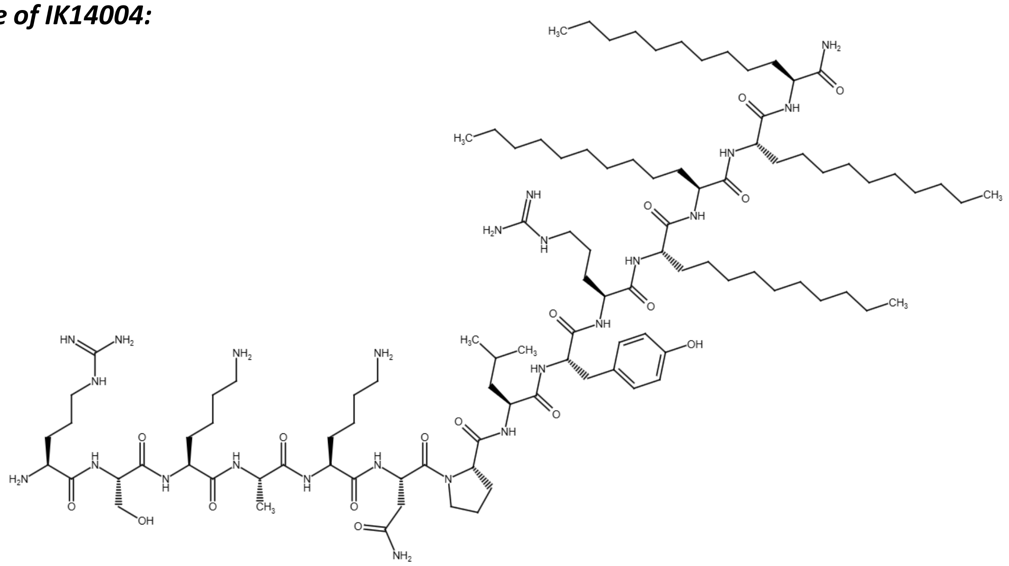

RP-HPLC purity: 99%.

Molecular Weight: Calculated for $C_{102}H_{186}N_{24}O_{17}$ (monoisotopic): 2019.44 amu. Found: 2019.80.

Amino Acid Analysis (AAA): Found: 1.03 x Asn, 0.99 x Ser, 1.92 x Arg, 1.00 x Ala, 0.99 x Pro, 0.98 x Tyr, 0.99 x Leu, 2.03 x Lys. *[(2)Adod not determined.]*

Peptide content by AAA: Calculated for the acetate salt, 87%,(w/w), Found 84% (w/w).

Figure S2: Refers to manuscript **Figures 1a and 1b**

IL-12Rβ2 (CD3+ T cells 72 hrs)

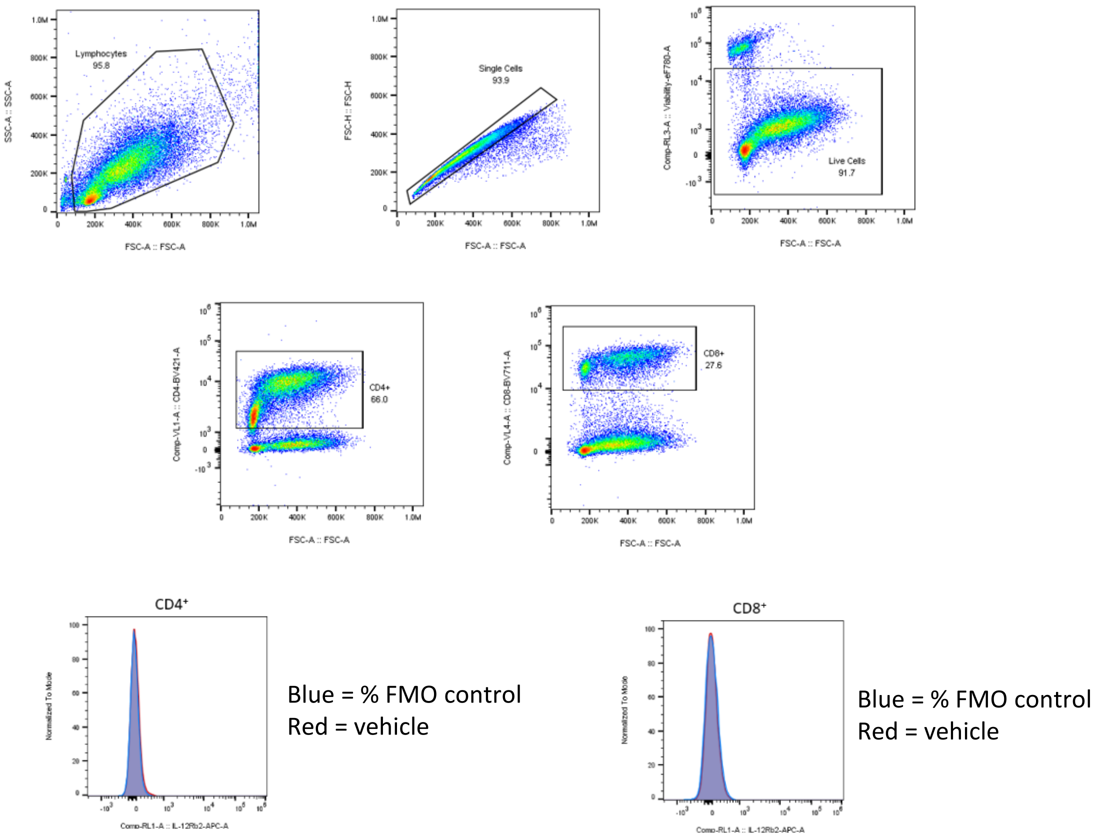

Representative gating strategy for the flow cytometric assessment of IL-12Rβ2 expression within the CD4+ and CD8+ T cell populations, within a stimulated CD3+ T cell assay. Cells were gated by forward and side scatter to remove debris, followed by doublet exclusion and selection of live cells. The blue histogram displays the % expression of the fluorescence minus one (FMO) control and red histogram the stimulated vehicle control.

Figure S3: Refers to manuscript **Figures 1c and 1d**

pSTAT3 (CD3+ T cells 72 hrs)

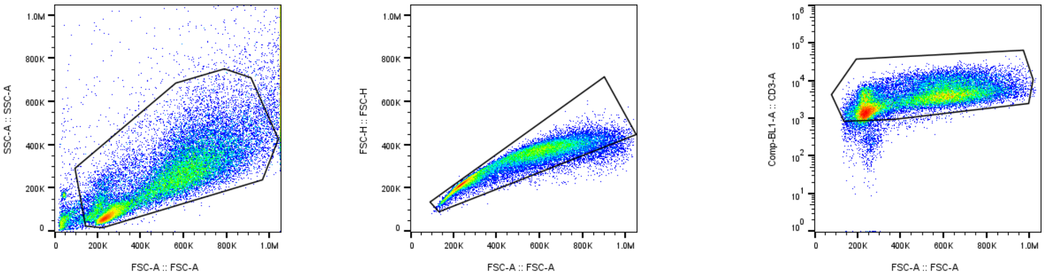

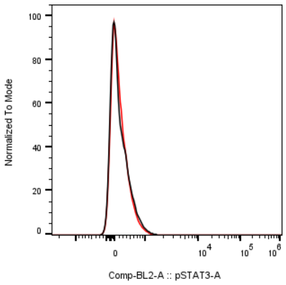

Grey = isotype
Black = vehicle
Red = IK14004 (1.25 µM)

Debris and multicellular events were excluded and CD3+ cells selected for analysis of pSTAT3

Figure S4: Refers to manuscript **Figure 1e**

pSTAT4 (CD3- CD56+ cells 24 hrs)

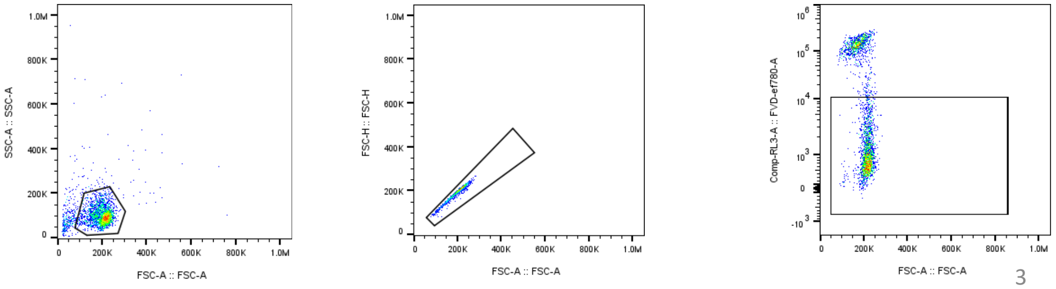

3

CD3- CD56+/ -pSTAT4

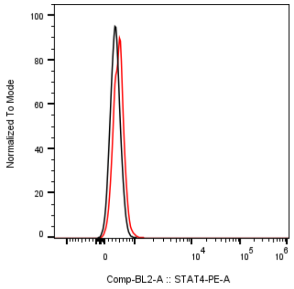

Grey = isotype
Black = vehicle
Red = IK14004 1.25 µM

Debris and multicellular events were excluded and viable (live) cells gated.

Figure S5: Refers to manuscript **Figures 1f and 1g**
Viability (CD3+ T cells 72 hrs)

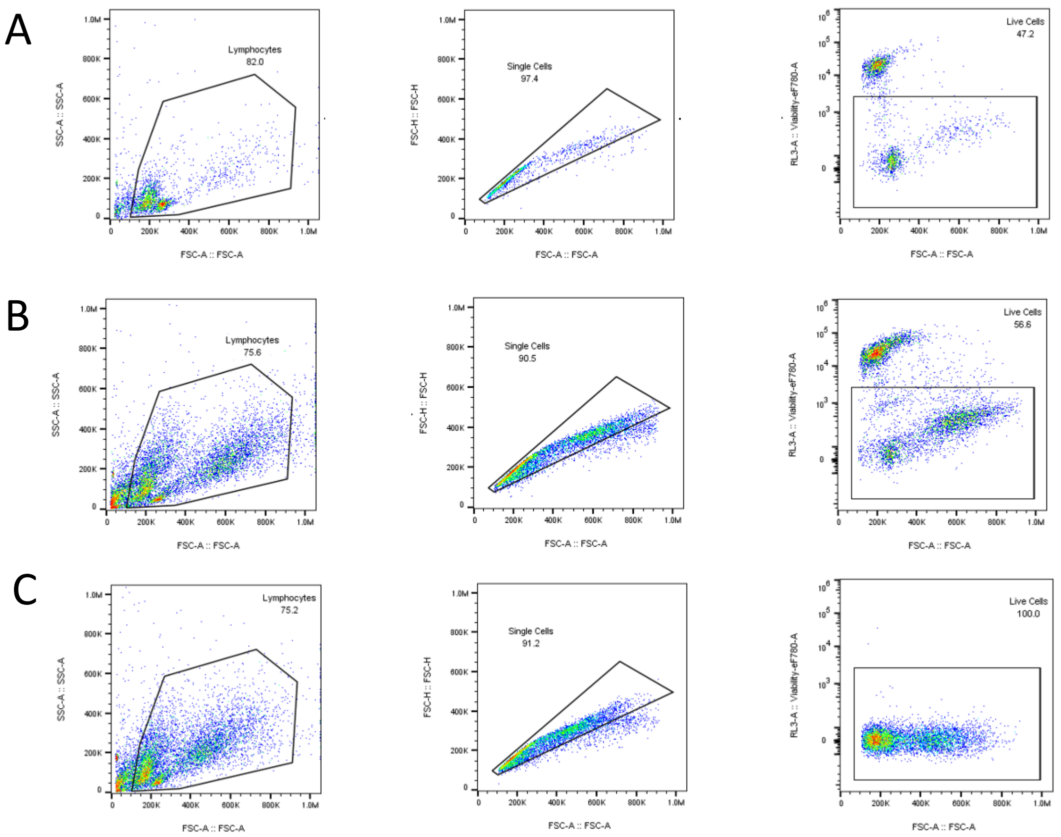

Representative gating strategy for the flow cytometric assessment of cell viability within a stimulated CD3+ T cell assay. Cells were gated by forward and side scatter to remove debris, followed by doublet exclusion and selection of live cells. (A) Represents the gating strategy to assess the proportion of live cells within the unstimulated control group, the stimulated control group (B) and unstained control group (C).

Figure S6: Refers to manuscript **Figures 1h and 1i**
pSTAT4 (CD3+ T cells 72 hrs).

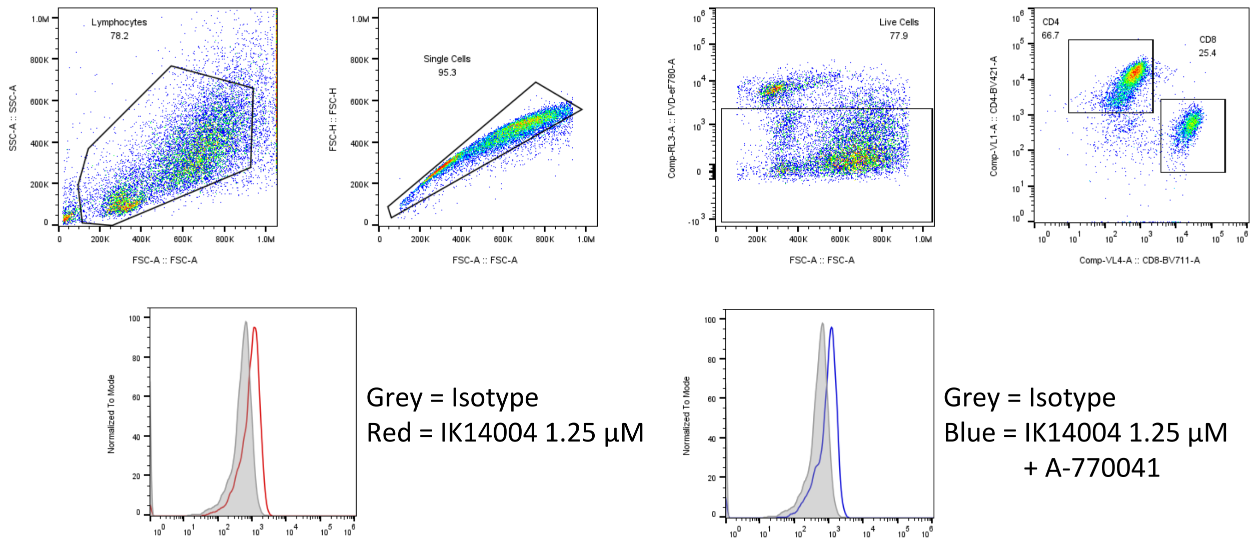

Representative gating strategy for the flow cytometric assessment of pSTAT4 within the CD4+ cell population, within a stimulated CD3+ T cell assay after 72 hrs. Cells were gated by forward and side scatter to remove debris, followed by doublet exclusion and selection of live cells.

Figure S7: Refers to manuscript **Figures 2a and 2b**
IFN-α and IFN-β (PBMC cultures 24 hrs)

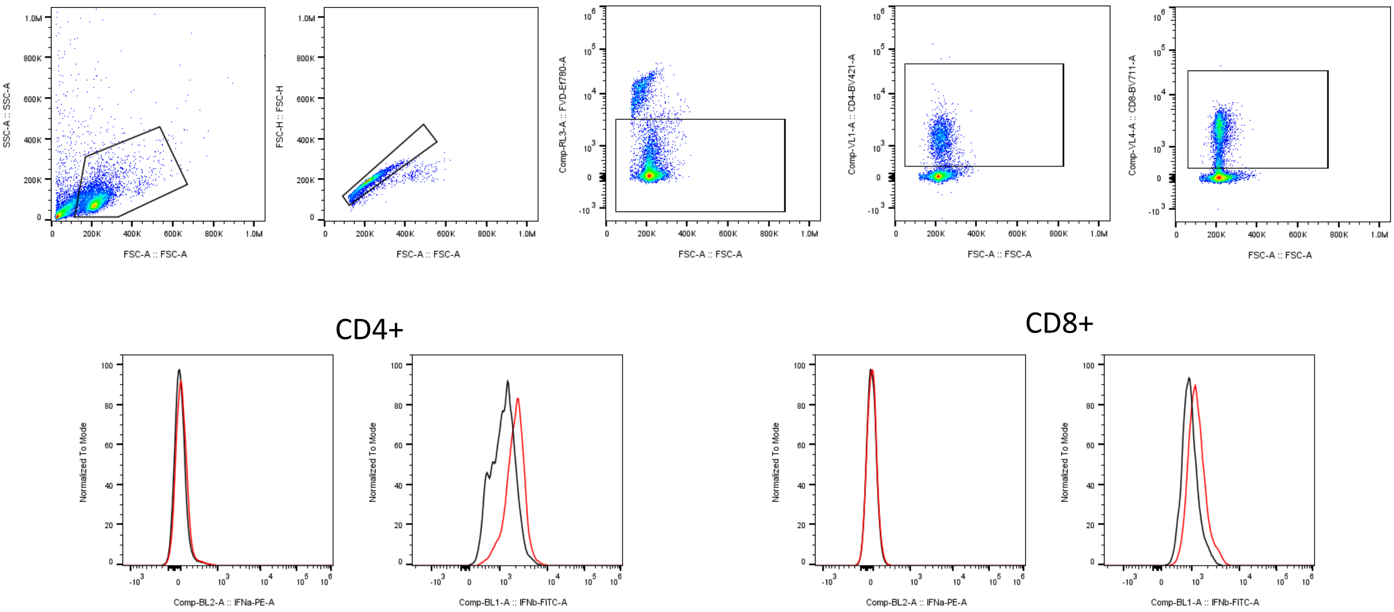

Grey = isotype
Black = vehicle
Red = IK14004 1.25 µM

Grey = isotype
Black = vehicle
Red = IK14004 1.25 µM

Representative gating strategy for the flow cytometric assessment of intracellular IFN-α and IFN-β expression within PBMCs. Cells were gated on size followed by doublet exclusion and selection of live cells. Within the viable cell population, CD4+ and CD8+ cells were selected and intracellular IFN-α and IFN-β expression determined within each population.

Figure S8: Refers to manuscript **Figures 2c and d**
IFN-α and IFN-β (CD3- CD56+ cells within PBMC cultures 24 hrs)

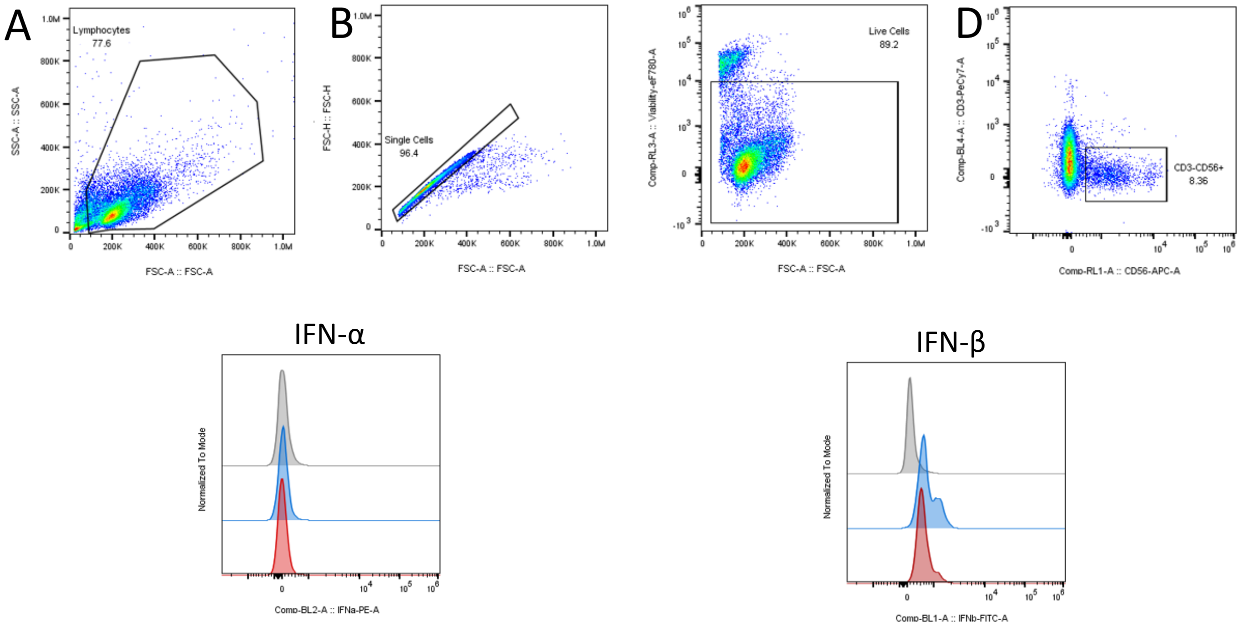

Cells were gated by forward and side scatter to remove debris (A), followed by doublet exclusion (B) and selection of live cells (C). Within the viable cell population, CD3- CD56+ NK cells were identified (D). The expression of intracellular IFN-α and IFN-β was then determined within CD3- CD56+ NK cells (E). The shift in % expression for intracellular IFN-α and IFN-β, is represented as a histogram overlay normalised to the mode. The grey histogram displays the % expression of the fluorescence minus one (FMO) control, blue histogram the unstimulated control and red histogram the stimulated vehicle control.

Figure S9: Refers to manuscript **Figure 3b**
CD215 (CD3- CD56+ cells within PBMC cultures 72 hrs)

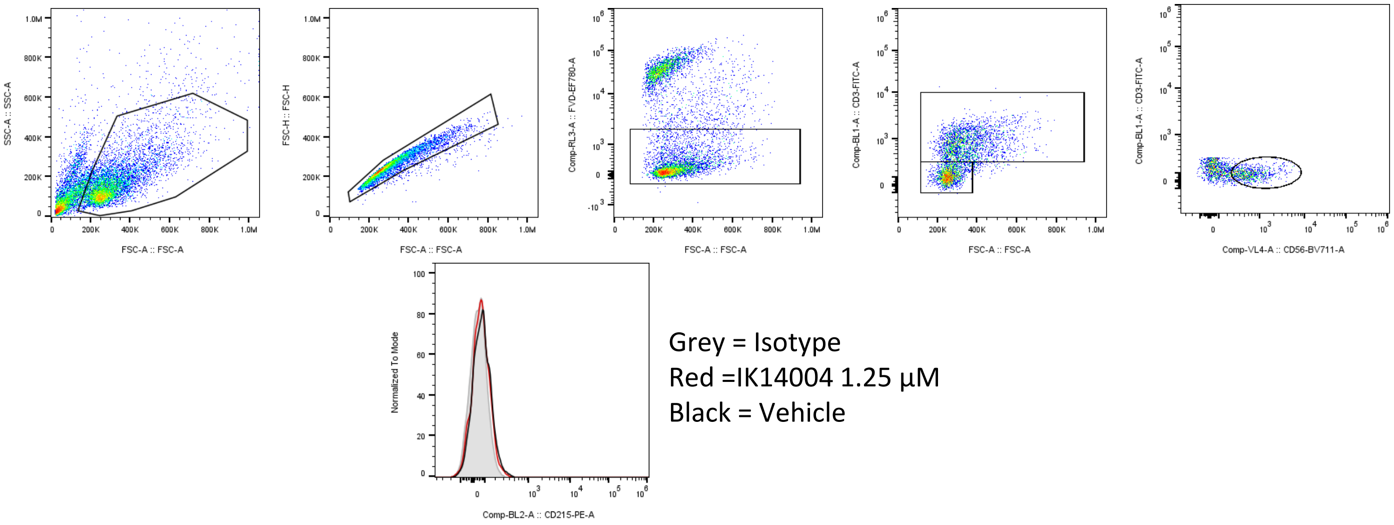

Debris and multicellular events were excluded, viable (live) cells gated and CD3- CD56+ cells selected for analysis of CD215.

Figure S10: Refers to manuscript **Figure 3c**
CD215 (Isolated CD3- CD56+ cells 72 hrs)

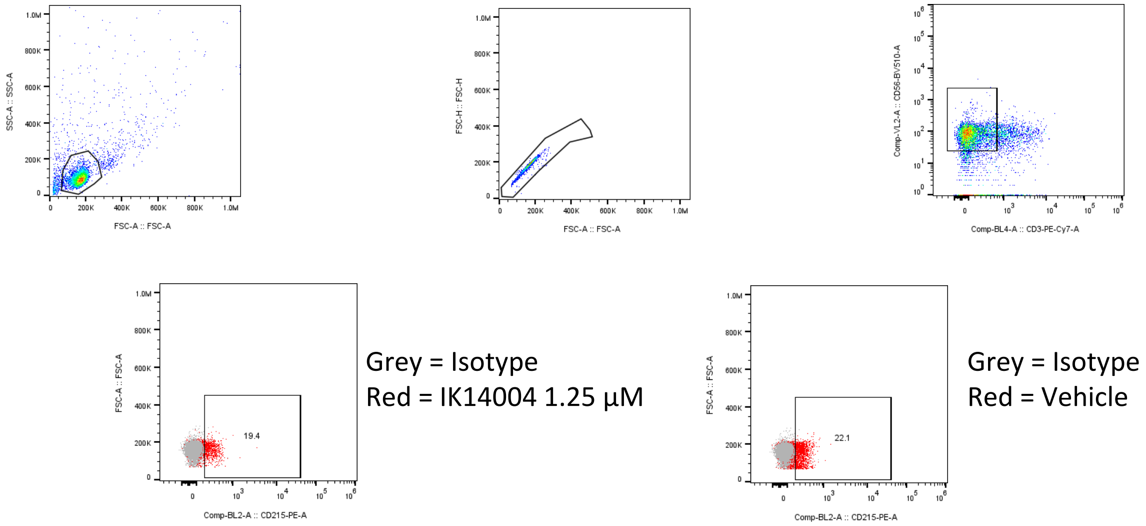

Debris and multicellular events were excluded and CD3- CD56+ cells selected for analysis of CD215.

Figure S11: Refers to manuscript **Figure 3d**
CD215 (CD3+ cells within PBMC cultures 72 hrs)

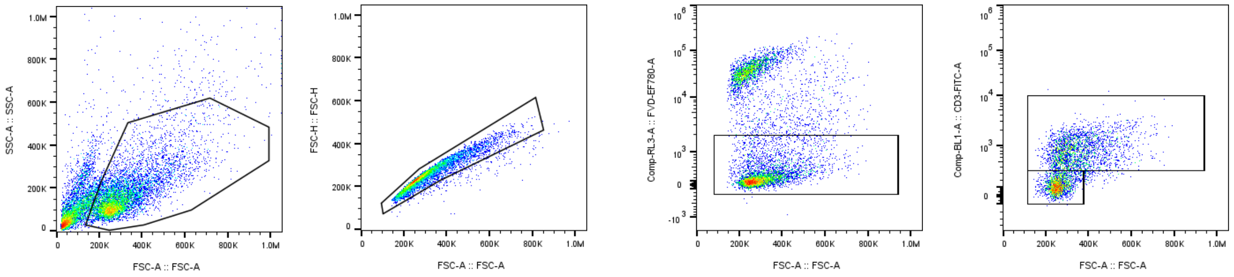

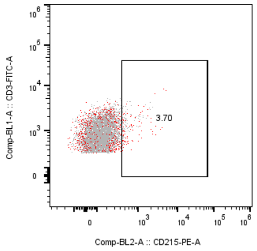

Grey = Isotype
Red = IK14004 1.25 µM

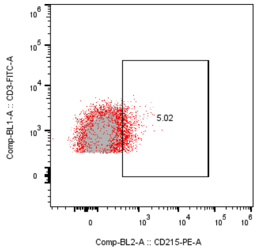

Grey = Isotype
Red = Vehicle

Debris and multicellular events were excluded, then viable (live) cells gated and CD3+ cells selected for analysis of CD215.

Figure S12: Refers to manuscript **Figures 3e-3h**
CD122 (CD4+/CD8+ cells within PBMC cultures 72 hrs)

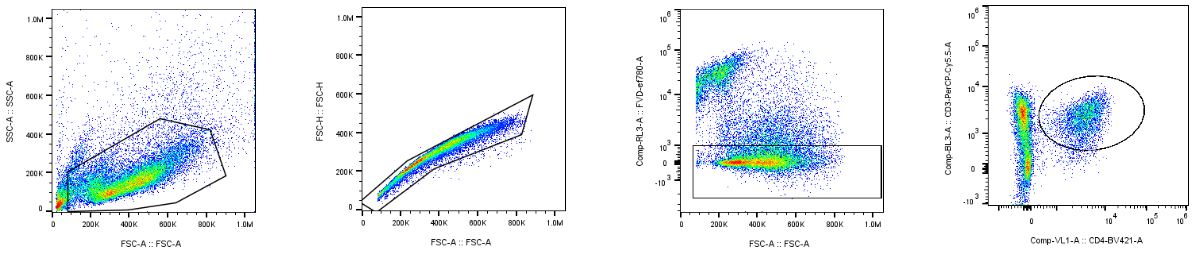

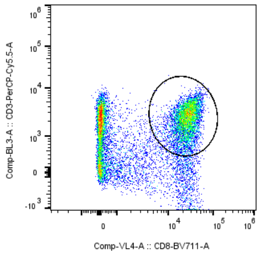

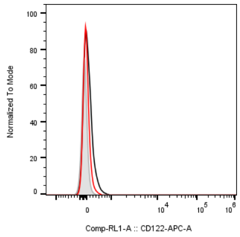

CD4⁺
Grey Isotype
Red IK14004 1.25 µM
Black Vehicle

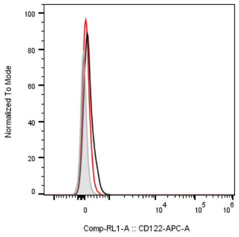

CD8⁺
Grey Isotype
Red IK14004 1.25 µM
Black Vehicle

Debris and multicellular events were excluded, then viable (live) cells gated and CD4+/CD8+ cells selected for analysis of CD122.

Figure S13: Refers to manuscript **Figures 3i and 3j**
CD122 (CD3- CD56+ cells within PBMC cultures 72 hrs)

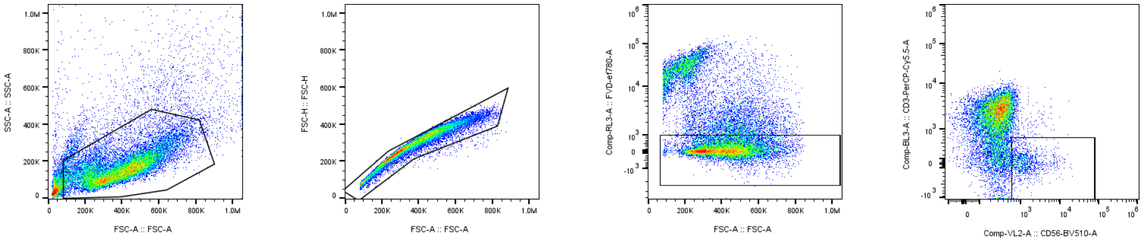

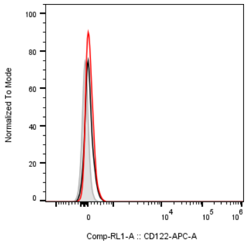

Grey = Isotype
Red = IK14004 1.25 µM
Black = Vehicle

Debris and multicellular events were excluded, then viable (live) cells gated and CD3- CD56+ cells selected for analysis of CD122.
